# Spanish version of the Heart Failure Somatic Perception Scale (HFSPS v.3) – psychometric properties

**DOI:** 10.3389/fcvm.2023.1242057

**Published:** 2023-12-01

**Authors:** Rosa Antonio-Oriola, Raúl Juárez-Vela, Michal Czapla, Angela Durante, Marco Di Nitto, José Vicente Benavent-Cervera, Carlos Saus-Ortega, Noelia Navas-Echazarreta, Ana Cobos-Rincón, Clara Isabel Tejada-Garrido, Ivan Santolalla-Arnedo, Vicente Gea-Caballero

**Affiliations:** ^1^ Doctorate Program in Clinical and Community Nursing, University of Valencia, Valencia, Spain; ^2^ Faculty of Health Sciences, GRUPAC Care Research Group, University of La Rioja, Logroño, Spain; ^3^ Department of Emergency Medical Service, Wroclaw Medical University, Wroclaw, Poland; ^4^ Department of Translational Medicine, Università del Piemonte Orientale, Vercelli, Italia; ^5^ Department of Health Sciences, University of Genoa, Genoa, Italy; ^6^ Faculty of Health Science, Research Group Community Health and Care, Valencia International University, Valencia, Spain; ^7^ Research Group in Art and Science in Care, Institute for Health Research La Fe (IISLAFE), University School of Nursing La Fe, València, Spain

**Keywords:** heart failure, signs, symptoms, psychometrics, reliability, validity

## Abstract

**Background:**

The Heart Failure Somatic Perception Scale (HFSPS) is an instrument that examine the existence and gravity of physical signs and symptoms in patients with heart failure, as well as early and subtle symptoms of HF that have clinical value, we aimed to translate and adapt the HFSPS from English to Spanish and evaluate the psychometric properties.

**Method:**

HFSPS translation and back translation were carried out according to the method established by of Beaton et al. A confirmatory factor analysis (CFA) was performed to test the factor structures. To assess criterion-related validity, HFSPS factor scores were correlated with Kansas City Cardiomyopathy Questionnaire (KCCQ) scores using the Spearman correlation method. The reliability of the internal consistency of the HFSPS was determined by calculating the Cronbach's alpha coefficient and the factor score determination coefficient.

**Results:**

Data from 173 patients with a mean age of 80.7 years (SD 9.1), women (51.1%), were analyzed. The majority (74.7%) were NYHA class II/III. The confirmatory factor analysis of four factors after eliminating one item showed fit indices close to the recommended indices: *χ*^2^ = 169.237, *p* < 0.001, CFI = 0.920, TLI = 0.901, RMSEA = 0.057 and SRMR = 0.061. Regarding the validity related to the criterion, all the scores of the HFSPS dimensions were correlated with all the scores of the KCCQ dimensions and were statistically significant. The reliability of the HFSPS factors of the coefficient of determination obtained scores of 0.73 for the dyspnea factor and early and subtle and lower for edema and chest discomfort with fewer items. Cronbach's alpha was acceptable for three of the scales >0.71 and poor 0.52 for chest discomfort with two items. The internal consistency index based on the model was 0.850.

**Conclusion:**

The Spanish version of the HFSPS is a valid and reliable instrument that that would be feasible to use in clinical and research setting to evaluate in the perception of symptoms in patients with heart failure.

## Introduction

Heart failure (HF) is a major health problem affecting more than 64 million people worldwide (1, 2). Although in recent decades, there has been evidence of an improvement in the prognosis of HF has improved slightly, death rate remains elevated, with a 1-year risk that varies between 15%–30% and 75% at 5 years. Heart failure is the main cause of hospital admission in patients older than 65 years of age and accounts for 1%–2% of all hospital admissions in western countries, with a high consumption of resources and healthcare costs. Thus, it has become an important global public health priority to contribute to establishing preventive strategies that lead to improved patient prognosis, such as understanding the causes of hospitalizations (2). Severity of dyspnea is an important prognostic marker in acute heart failure. Approximately 50% of patients with acute heart failure and dyspnea on hospital admission reported dyspnea at rest which is associated with increased mortality, readmission, hospital stay and costs (3).

It is problematic for the patient to detect and attribute significance to early symptoms of HF decompensation, so response in seeking timely care is inadequate. Nearly half of patients have dyspnea for three days or more prior to hospitalization as a result of poor symptom detection (4). Less acute symptoms of fatigue, cough, edema, and weight gain, which patients might potentially consider to be a consequence of aging or other less threatening illnesses, are tolerated for relatively prolonged periods delaying attention until these interfere with their daily activities (5, 6).

Symptoms are multidimensional and include mental, emotional components, and physical aspects so the process of symptom perception by patients results in highly variable and often inaccurate interpretations of symptom meaning, which influences physicians' recognition of the multiple signs and symptoms that comprise the diagnostic criteria for heart failure (4, 7, 8). Thus, there is a need for reliable and valid tools that systematically record patients' assessment of symptom perception that would help predict the risk of morbidity and mortality and prevent potential adverse outcomes (9, 10).

Measures that have been investigated to Identify and link common symptom profiles to survival vary in both method, levels of completeness of measures, as well as in the type, number of symptoms and dimensions assessed (11–14). In addition, several of the instruments have limitations by not assessing early and subtle indicators of HF decompensation or measure with a single item dyspnea, a symptom that varies in intensity according to disease activity and severity and can help identify early decompensation (9).

The European Society of Cardiology guidelines for HF recommend self-care strategies to reduce the risk of HF hospitalization and mortality, with approaches in which patients and caregivers take an active role, with collaborative communication and activities. Self-care and symptom management is one of the components of heart failure care programs, which they consider key, where the patient should act by monitoring and recognizing changes in signs and symptoms, reacting appropriately to changes, and knowing how and when to contact a healthcare professional (15). In order to accurately quantify the full spectrum of, symptom experienced by patients it is necessary to use symptom questionnaires created specifically for patients with heart failure (16).

The HFSPS was developed based on Lenz's Unpleasant Symptom Theory regarding the interactions between multiple symptoms, the multiple physiological and psychological mechanisms that influence symptom perception, situational factors that relate to personal experiences, and the outcomes or effects of symptom experience (17, 18). The original Heart Failure Somatic Perception Scale (HFSPS) (19), included 12 items corresponding to 12 physical symptoms of heart failure. Because it failed to consider complexity of dyspnea among others symptoms of heart failure, Jurgens et al. increased the scale from 12 to 18 items to capture the more subtle symptoms of HF, adding exertional dyspnea, fatigue, nocturia, and symptoms related to right-sided congestion (abdominal bloating in the abdomen and appetite loss). The HFSPS determines whether signs and symptoms are present and how severe they are and dyspnea and their effects on daily activities (9). In addition, The five criteria put forth by Lee and Moser are reflected in the HFSPS (13) to assess the quality of self-reported symptom measures created and utilized in patients with heart failure: (1) content, including number and description of symptoms as well as the dimensions assessed in terms of prevalence, frequency, severity and distress; (2) measurement scale, simple and ideal for clinical and research use, and simple to complete; (3) psychometric properties, an accurate (internal consistency and test-retest reliability) and precise instrument (content, criterion and construct validity) (4) completion process, burden and time spent (5) Information on clinical consequences associated with prognosis as in the case of survival and quality of life.

In the psychometric analysis of the HFSPS by Jurgens et al. and Pucciarelli et al. (9, 20) showed that the HFSPS is a valid and reliable instrument to measure the physical signs and symptoms of patients with heart failure in the dimensions of dyspnea, chest discomfort, early and subtle and edema, with a robust dyspnea subscale that explores a wide range and degree severity of dyspnea symptoms being effective in predicting clinical events related to HF, as well as assessing early and subtle symptoms of HF that have clinical value (21–23).

Having an instrument with these characteristics in Spain, where the level of self-care is significantly lower compared to other countries regarding the management of their disease (24) is essential to to evaluate how complex of heart failure symptoms to be able to identify patients at risk.

Therefore, the purpose of this study was to translate and adapt into Spanish and evaluate the psychometric properties of the HFSPS in a population of Spanish patients with heart failure.

## Methods

### Design

This is a complementary analysis of an observational descriptive transversal study with all measures administered at one point at a time, with the aim of determining the level of self-care performed in a population of patients with heart failure and their caregivers, as well as determining the level of understanding and ability to recognize signs and symptoms, with the purpose of evaluating and improving self-care in patients with Heart Failure. This study followed two separate phases: (1) cross-cultural adaptation of the HFSPS v.3 and (2) test of psychometric properties of the HFSPS v.3.

### Study sample and setting

This study was carried out in the Hospital Clinico de la ciudad Española de Zaragoza in 2017. The inclusion criteria were: (1) receive a diagnosis of HF based on the criteria of the European Society of Cardiology (ESC) criteria (15), and (2) Be over 18 years old. Patients with important cognitive impairment determined by obtaining with the Six-Item Screener score of less than 4 points scale for the detection of cognitive impairment were excluded (25).

### Adaptation, translation and modeling

Its psychometric capacities were measured before carrying out the cross-cultural adaptation process of the HFSPS v.3 (9). It was translated and adapted from its original English version into Spanish conforming steps of the protocol to Beaton et al. (26), This breaks the procedure down into six steps: (1) forward translation, (2) summary translations, (3) reverse translation, (4) competent adviser committee verification, (5) checking of the preliminary version, and (6) Presentation documentation to the Evaluation Committee that monitors the Process.

According to this methodology, the original HFSPS v.3 was translated into Spanish by a bilingual researcher who was versed with him concepts of the questionnaire. In order to develop a more reliable equivalence, the two translators received guidelines to avoid hypothetical questions, metaphors, informal language and other types of sentences that are not simple ones. To find out conceptual equivalence, the reverse back translation version of the HFSPS v.3 was reviewed by the original instrument's creators, and a finished, final version in Spanish was created in Spanish was established, but not before minor adjustments they were solved. Subsequently, a committee of experts made up of health professionals, translators involved in the process and native teachers with clinical practice in both languages and knowledge in research methodology, who debated compared the back-translated text to the original HFSPS v.3 and decided on a final version by consensus in Spanish. The aim of the expert committee was to Spanish version of the HFSPS v.3 closest match possible to the original language.

Finally, It was completed with cognitive interviews with a convenience sample of 32 patients to evaluate compressibility and applicability of the Spanish version.

### Measures

The HFSPS v.3 (9), which consists of 18 items that measure the physical signs and symptoms of heart failure patients that that disturbed the patient in the last seven days, composed of four dimensions: dyspnea composed of six items, chest discomfort by two, early and subtle by seven and edema by three items, using 5 response options ranging from 0 (did not have the symptom) to 5 (extremely bothersome). Scores are calculated by summing all the elements, with a amplitude from 0 to 90 Higher values indicate a greater symptom burden in the patient's life. The Kansas City Cardiomyopathy Questionnaire (KCCQ) (27) adapted to Spanish (28) is a 23-item self-administered health status-specific quality-of-life instrument for patients with chronic heart failure, composed of seven dimensions: physical limitation; symptoms (stability, frequency, and severity); quality of life; social limitation; and self-care. The KCCQ is a valid and reliable measure of state of health that includes an assessment of changes in symptoms and level of self-care. It is Likert-type and the score of each of its dimensions ranges from 0 to 100, with 100 being the best health status (quality of life), with two summary scores: (1) the functional status which is the sum of the physical and symptom limitation score excluding symptom stability; and (2) the clinical summary, combining the functional status with the domains of quality of life and social limitation. The KCCQ was used for criterion validity because physical symptom burden are strong predictors of poor quality of life (29).

#### Data collection procedure

Once patients gave informed consent to participate in the study, they were interviewed during admission in the period from February to December 2017, by qualified nurses who had been trained specifically for this project, A questionnaire with sociodemographic (age, sex, marital status, educational level and current job) and clinical (time with HF, NYHA functional class, Ejection Fraction and HF Etiology) data was also collected.

#### Data analysis

Descriptive statistics, which included means, standard deviations (SD), frequency and percentages, was used to describe the patients' sociodemographic and clinical variables as well as to describe the measurements.

Means, SD, and normal distribution were calculated with skewness and kurtosis indices of the HFSPS items. A value of skewness and kurtosis between −1 and 1 is generally considered a slight deviation from normality (30).

The Kaiser–Meyer–Olkin measure of sampling adequacy and Bartlett's test of sphericity were used to assess the factorization of the data. Significant results of Bartlett's test of sphericity (*p* < 0.0001) and KMO index >0.50 indicate that it is relevant to use factor analysis with this sample (31). The measurement variables' descriptive statistics and correlation matrices were then examined to rule out any issues with multicollinearity or missing data. A confirmatory factor analysis (CFA) was performed to test the factor structures of the HFSPS published by Jurgens et al. of four factors: dyspnea that groups the items 2, 7, 9, 12, 13 and 17, chest discomfort items 1 and 3, early and subtle that groups the items 4–6, 14–16, and 18, and edema with the items 8, 10, and 11. For the analysis, the Varimax rotation method with Kaiser and the principal component estimation method were used. The implicit correlations of the model were also estimated whose value should not be >0.70.

To examine the adequacy of the model tested, a multifaceted approach was adopted and the following indices and fit criteria were evaluated: *χ*^2^ test non-significant values are interpreted as supporting model fit; comparative fit index (CFI) and Tucker–Lewis index (TLI), which values of >0.90 indicate a good fit; root mean square error of approximation (RMSEA), which values of <0.05 indicate a good model fit; and Standardized root mean square residual (SRMR) which values of <0.08 indicate a good sample fit (32). Using Spearman's correlation method, HFSPS factor scores were correlated with KCCQ scores to assess criterion-related validity (33). The internal consistency of the HFSPS was determined by calculating Cronbach's alpha coefficient (>0.70) (34) and the coefficient of factor score determination, as in Jurgens et al. (9). The total score of the instrument as well as the calculation of each dimension was considered.

The analysis of the data was carried out with out with IBM SPP-AMOS V24 and SPSS statistics. (IBM Corporation, New Orchard RD Armonk, NY, EEUU).

## Ethical considerations

The study protocol was reviewed and approved before the registers collection began by a local research ethics committee (reference number P15/0216). Before enrollment, each participant obtained information about the objectives of the study and provided their written consent. Free participation and privacy protection guaranteed.

## Results

Table 1 illustrates the main sociodemographic characteristics of the sample. The mean age was 80.7 years. There were slightly more women than men (51.1%), widowed (47.1%), pensioners (94.3%), and primary school educated (86.8%). The main etiology of heart failure was non-ischemic cardiomyopathy (54%). Most participants (74.7%) had NYHA class II/III symptoms.

**Table 1:** Main sociodemographic characteristics of the sample (*n* = 173).

Variables	*n*	(%)
Age (mean, SD)	80.7	(9.10)
Gender		
Men	85	(48.9)
Women	88	(51.1)
Civil status		
Single	12	(6.9)
Married	78	(45.4)
Separated/divorced	1	(0.6)
Widower	82	(47.1)
Education level		
Primary education	150	(86.8)
Secondary education	11	(6.3)
Vocational training	1	(0.6)
Baccalaureate	5	(2.9)
University studies	6	(3.4)
I currently work		
Employee	3	(1.7)
Self-employed	4	(2.3)
Pensioner	164	(94.3)
Unemployment	2	(1.7)
Time with heart failure, months (*n* = 127)	3.76	(1.8)
Functional classification NYHA (*n* = 133)		
Class I	1	(0.6)
Class II	66	(37.9)
Class III	64	(36.8)
Class IV	2	(1.1)
Ejection fraction % (*n* = 48)	47.41	(14)
Etiology of heart failure (*n* = 129)		
Ischemic	33	(19)
Non ischemic	94	(54)
Idiopathic (unknown cause)	2	(1.1)
Others		

The total of the sample is the one that appears in the description of the table. The parentheses that appear for each variable refers to the number of complete cases.

### Descriptive analysis of the HFSPS items

The mean, standard deviation (SD), skewness, and kurtosis values for the Spanish version of the HFSPS of the items are reported in Table 2. The means of the item scores ranged from 0.69 to 3.91. The items with the highest means were “I felt tired” and “It became difficult to breathe”; the items with the lowest means were “I had stomach discomfort” and “My clothes feel tighter around my waist” from the early and subtle dimension. The skewness and kurtosis indices showed that not all items followed a perfect normal distribution (Table 2).

**Table 2:** Heart failure somatic perception scale item descriptive (*N *= 173)**.**

Items	Mean	SD	Skewness	Kurtosis
1. I could feel my heart beat get faster	1.55	2.03	0.70	−1.29
2. I could not breathe if I lay down flat	2.26	2.14	0.05	−1.77
3. I felt discomfort or pain in my chest	1.27	1.93	1.06	−0.64
4. I had an upset stomach	0.69	1.41	1.91	2.26
5. I had a cough	1.09	1.71	1.21	−0.10
6. I was tired	3.91	1.67	−1.51	0.92
7. I could not catch my breath at the end of the day	2.76	2.11	−0.29	−1.64
8. My feet were swollen	2.09	2.14	0.25	−1.70
9. I woke up at night because I could not breathe	1.30	1.87	0.97	−0.73
10. My shoes were tighter than usual	2.10	2.15	0.27	−1.68
11. I gained weight in the past week	1.58	1.96	0.71	−1.14
12. I could not do my usual activities because I was SOB	1.94	1.98	0.31	−1.55
13. Getting dressed made it hard to breathe	2.14	1.97	0.10	−1.62
14. My clothes felt tighter around my waist	1.01	1.62	1.21	−0.14
15. I woke up at night because I had to urinate	1.72	2.04	0.53	−1.46
16. I had to rest more than usual during the day	1.95	1.97	0.26	−1.57
17. It was hard for me to breathe	3.05	2.01	−0.54	−1.33
18. I did not feel like eating	1.15	1.70	1.04	−0.50

SOB, short of breath.

#### Confirmatory analysis of HFSPS

The CFA test of the original four-factor model identified by Jurgens et al. (9) showed unsatisfactory fit indices: *χ*^2^ = 302.135, *p* < 0.001, CFI = 0.792, TLI = 0.757, RMSEA = 0.083 y SRMR = 0.083. The correlations between the items of the scale obtained adequate values since they did not exceed the recommended value (Table 3).

**Table 3:** Implied correlations (model HFSPS).

	11	10	8	18	16	15	14	6	5	4	3	1	17	13	12	9	7	2
11	1																	
10	0.339	1																
8	0.394	0.575	1															
18	0.193	0.281	0.327	1														
16	0.175	0.256	0.298	0.434	1													
15	0.16	0.234	0.272	0.396	0.36	1												
14	0.197	0.287	0.334	0.486	0.443	0.404	1											
6	0.039	0.057	0.066	0.097	0.088	0.08	0.099	1										
5	0.115	0.168	0.196	0.285	0.26	0.237	0.291	0.058	1									
4	0.139	0.202	0.235	0.343	0.312	0.285	0.35	0.07	0.205	1								
3	0.149	0.217	0.253	0.288	0.263	0.24	0.294	0.059	0.173	0.208	1							
1	0.174	0.254	0.296	0.337	0.307	0.28	0.344	0.069	0.202	0.243	0.348	1						
17	0.074	0.108	0.126	0.143	0.13	0.119	0.146	0.029	0.086	0.103	0.111	0.129	1					
13	0.157	0.229	0.267	0.304	0.277	0.253	0.311	0.062	0.182	0.219	0.235	0.275	0.152	1				
12	0.181	0.264	0.307	0.35	0.319	0.291	0.357	0.071	0.21	0.252	0.27	0.316	0.175	0.371	1			
9	0.169	0.246	0.286	0.327	0.298	0.272	0.334	0.066	0.196	0.235	0.253	0.295	0.163	0.347	0.399	1		
7	0.154	0.224	0.261	0.298	0.271	0.247	0.304	0.06	0.178	0.214	0.23	0.269	0.149	0.316	0.363	0.339	1	
2	0.16	0.233	0.271	0.309	0.282	0.257	0.316	0.063	0.185	0.223	0.239	0.279	0.154	0.328	0.377	0.352	0.321	1

Bartlett's test of sphericity for the entire questionnaire was significant (*p* < 0.001), and the KMO sample adequacy index was 0.828. The data were adequate to perform factor analysis based on the results.

Initially, an exploratory factor analysis was performed which reflected the internal structure of the questionnaire items and factors, and after adjusting the weights of the items, item 6 was removed, not without first reviewing the modification indices to assess a possible mismatch between the items. Subsequently, a confirmatory factor analysis was performed using the original model extended by Jurgens et al. in 2017 of four factors which showed fit indices that were close to the recommended indices: *χ*^2^ = 222.503, *p* < 0.001, CFI = 0.858, TLI = 0.832, RMSEA = 0.074 y SRMR = 0.070. Given these results, the modification indices were reviewed again, which showed excessive covariance, between the residues of items 15 and 16 of the same dimension (which examined the need to rest during the day and nocturia), items 5 and 8 (which investigated swollen feet at the end of the day with cough), items 17 and 15 (which examined difficulty breathing with nocturia) and items 7 and 13 of the same dimension (who examined difficulty catching one's breath when dressing).By allowing the residuals of the items to correlate, satisfactory fit indices were obtained: *χ*^2^ = 169.237, *p* < 0.001, CFI = 0.920, TLI = 0.901, RMSEA = 0.057 y SRMR = 0.061.

The 18 items of the questionnaire were represented by weights ranging from 0.04 for item 6 (I felt tired) to 0.85 for item 10 (My shoes were tighter than usual).Only one item (item 6: I felt tired) of the early and subtle dimension saturated below 0.30 whose value was 0.04, so it was eliminated by confirming in the analysis that it had less factor loadings. The result of the variance explained improved the result of the subscale (39.4%–46%) and of the questionnaire (54.5%–55.2%), as well as Cronbach's alpha, which went from 0.70 to 0.76 early and subtle subscale.

**Figure 1:**
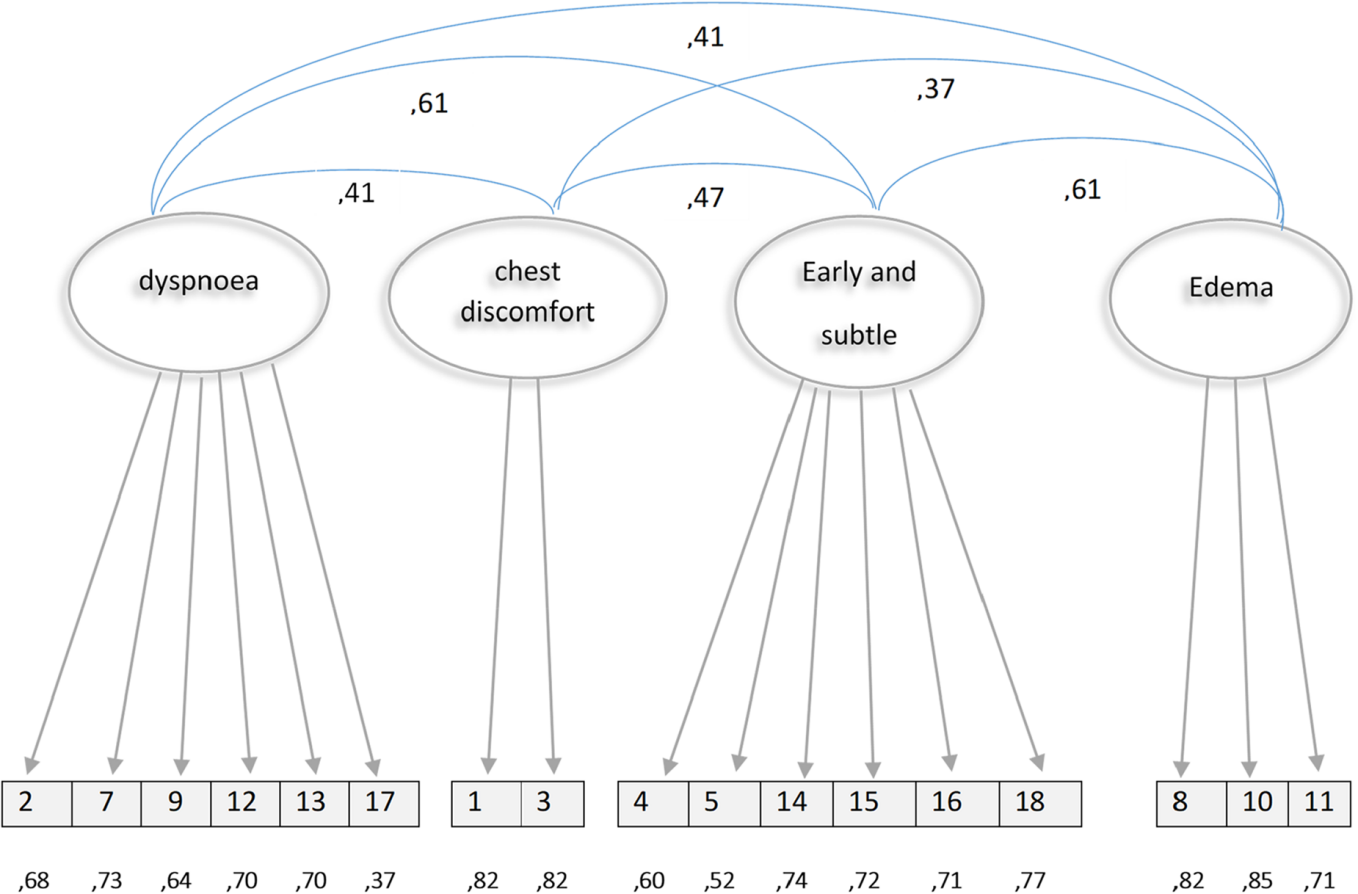
Graphic representation of the confirmatory factor analysis of the HFSPS.

All the factor loadings of the 17 items were statistically significant, with values between 0.37 for item 17 and 0.85 for item 10. The correlations between the four factors were also statistically significant, ranging from 0.37 for the chest discomfort and edema factor to 0.605 for the early and subtle factor and the dyspnea factor (9). Figure 1 shows the graphical representation of the CFA.

### Criterion validity related to the HFSPS

Using Spearman's Rho, the criterion validity was examined correlating the four HFSPS components with the KCCQ dimensions. Were statistically significant all correlations, ranging from −0.580 among the HFSPS total score and the total of the symptom scores obtained from the KCCQ −0.171 among the HFSPS chest discomfort dimension and the KCCQ symptom stability scores (Table 4).

**Table 4:** Correlations between the KCCQ and the HFSPS.

	1	2	3	4	5	6	7	8	9	10	11	12	13	14	15
1. Physical limitation KCCQ	1														
2. Stability symptoms KCCQ	.335 **	1													
3. Symptom frequency KCCQ	.512 **	.436 **	1												
4. Charge symptoms KCCQ	.501 **	.481 **	.872 **	1											
5. Total symptoms KCCQ	.541 **	.565 **	.966 **	.952 **	1										
6. Quality of life KCCQ	.536 **	.273 **	.481 **	.461 **	.494 **	1									
7. Social limitation KCCQ	.508 **	.212 **	.435 **	.403 **	.437 **	.960 **	1								
8. Self care KCCQ	.488 **	.432 **	.935 **	.864 **	.924 **	.473 **	.431 **	1							
9. Clinical summation KCCQ	.836 **	.478 **	.877 **	.858 **	.902 **	.594 **	.547 **	.836 **	1						
10. General summation KCCQ	.785 **	.427 **	.789 **	.763 **	,808 **	.851 **	.819 **	.764 **	.917 **	1					
11. Score HFSPS	−.439**	−.424**	−.515**	−.532**	−.560**	−.462**	−.400**	−.514**	−.580**	−.578**	1				
12. Dyspnoea HFSPS	−.366**	−.312**	−.490**	−.472**	−.510**	−.471**	−.416**	−.507**	−.513**	−.545**	.857 **	1			
13. Chest discomfort HFSPS	−.199**	−.171*	−.230**	−.282**	−.269**	−.397**	−.363**	−.258**	−.283**	−.364**	.612 **	.410 **	1		
14. Early and Subtle HFSPS	−.370**	−.397**	−.317**	−.349**	−.365**	−.352**	−.283**	−.331**	−.418**	−.416**	.844 **	.605 **	.468 **	1	
15. Edema HFSPS	−.366**	−.424**	−.468**	−.509**	−.525**	−.264**	−.229**	−.401**	−.518**	−.449**	.668 **	.407 **	.369 **	.416 **	1

* The correlation is significant at the 0.05 level (bilateral).

** The correlation is significant at the 0.01 level (bilateral).

### Reliability of the HFSPS

The reliability of the HFSPS factors was evaluated by calculating the coefficient of determination, which resulted in: 0.73 for dyspnea, 0.73 for early and subtle factor, 0.45 for edema and 0.44 for chest discomfort. Cronbach's alpha was used to evaluate the internal reliability of the HFSPS questionnaire, which was acceptable for three of the scales: 0.72 for dyspnea, 0.76 for early and subtle, 0.71 for edema and a poor 0.52 for chest discomfort. For the full scale, the model-based internal consistency index was 0.850.

## Discussion

This study's objective was to perform the cross-cultural adaptation and validation of the HFSPS and to evaluate the psychometric properties in a population of Spanish patients with heart failure. The results showed a reliable and valid to measure physical symptoms and signs of patients affected by heart failure. This study is the first to validate the HFSPS in Spanish that we are aware of. So far only one similar validation study has been conducted in a European country, in Italy, evaluating the psychometric properties of the HFSPS (20). Small adjustments were made to the translation during the cross-cultural process, to enhance the items and to ensure cultural equivalence.

Regarding participant characteristics, the mean age in our study was 80.7 years, which differs from the sample used by Jurgens et al. when they extended the scale in 2017 which was 62.6 years (9). The sample was the oldest compared to the original construct (70 years) (17) and to the Italian validation (71.48 years) (20), which may represent a difference for the study in answering the early and subtle scale items because of the difficulty in recognizing the subtle and non-specific symptoms of heart failure, and consider a consequence of aging (4–6).

Initially the CFA not supported the original model of Jurgens et al. (9) of four factors: “dyspnea”, “chest discomfort”, “early and subtle” and “edema”, where unsatisfactory fit indices were shown. The values obtained in the implicit correlations in the model were adequate since they did not exceed the recommended value.

The 18 items obtained high scores in general, with the exception of item 6 “I felt tired” of the early and subtle dimension that saturated with a very low value (0.04), so it was decided to eliminate it when it was confirmed in the analysis that it had less factor loadings, improving the explained variance. A possible explanation is that it could be related to the age (80.7) of our respondents because patients consider it to be attributed to aging (4–6).

Subsequently, a confirmatory factor analysis was carried out, which showed fit indices close to the recommended indices. All factor loadings of the 17 items were statistically significant as well as the correlations between the four factors.

All four scales showed generally high factor loadings, but the one for chest discomfort and edema were higher. The initial setting of the Italian study (20) was also unsatisfactory. They found satisfactory fits by allowing the residuals of some of the elements to correlate with freedom, but this was not our case, since it did not allow us to correlate items with similar content, therefore it was decided to eliminate an item.

Once eliminated, the fit indices were reviewed as in the Italian article, finding a satisfactory fit. The residuals of the items that were freely correlated reflected theoretically similar content. For example, items 15 and 16 of the same dimension “Early and Subtle” reflected the need for daytime rest and nocturia the onset of fluid retention, items 17 and 15 that reflected difficulty breathing, of the dimension “ Dyspnea” and nocturia from the “Early and Subtle” dimension, the most advanced fluid retention, as well as items 5 and 8 with swollen feet at the end of the day from the “Edema” dimension and cough from the “Early and Subtle” reflected the edema. Finally, with items 7 and 13 of the same dimension “Dyspnea” they reflected the difficulty to catch one's breath with the action of dressing. It is appropriate to allow the residuals of the items to be correlated in a CFA as pointed out Bagozi (35). Pointed out, provided that these correlations are conceptually or methodologically possible and their estimates do not affect the estimated values of the parameters that make up the model. Regarding the model of Jurgens et al. (9), the fit indices were not perfect, but they were very good in most of the metrics analyzed, as revealed by the analyzes of our study.

The results of the analysis supported the criterion-related validity of the HFSPS. All the scores of the HFSPS dimensions and the KCCQ were correlated and were statistically significant but moderate as in the Italian study (20). These results support those obtained by other studies showing that quality of life as measured by the KCCQ correlates with Heart Failure symptoms (36).

For reliability we replicated the same tests as the Italian study. Factor score coefficients of determination were adequate for the “dyspnea” and “early and subtle” scales. They were lower for “edema” and “chest discomfort” with fewer items, a result that differed from the Italian study.

On the other hand, Cronbach's alpha obtained similar values that were acceptable for all the scales but poor 0.52 for the “chest discomfort” scale with only two items. According to the literature Cronbach's alpha can be lower when the scale is composed of fewer items (37). As recommended by Jurgens et al., the internal consistency index was used to calculate the reliability of the entire multidimensional scale (37) and the Italian study (20). The results were similar supporting the reliability of the HFSPS for the whole scale. All the scores of the HFSPS dimensions and the KCCQ were correlated.

Currently, In Spain there is no scale that measures signs and symptoms of heart failure, effective in the prediction of clinical events related to HF. The HFSPS is a tool that can give support medical staff to assess the complexity of heart failure symptoms in order to identify patients at risk, allowing the elaborate of individual plans directed at helping the patient to recognize changes in signs and symptoms and to react appropriately. This information can have repercussions on health, quality of life, hospital admissions and, by extension, it is a measure that provides economic benefits to the health system.

In the future, and in order to study new versions of the questionnaire, it would be important to test the complete questionnaire with samples with a mean age similar to that of other studies to assess the loads of the deleted item. This would allow the use of different models depending on the differential functioning of the items.

## Limitations

It is important to underline some of the limitations of the study. Although sufficient to meet the objectives of the study, the sample size is lower in number used in the original version as well as in the Italian adaptation.

The age of the sample would be another limitation because there are a series of symptoms that are inherent to age. It could also be an advantage in examining differential item functioning.

Another limitation is the kind of sampling that was employed in the study which could generate the appearance of a Berkson bias, since it was tested in recently hospitalized patients; therefore, it would be possible that they were the ones with the most developed disease. However, and considering the trend of admission and readmission of patients with heart failure, we consider the probability of the appearance of bias to be remote.

## Conclusion

Our research has shown revealed that the Spanish version translation of the HFSPS is a valid and reliable instrument that that would be feasible to use in clinical IC and research setting to evaluate in the perception of symptoms in patients with in the dimensions of dyspnea, chest discomfort, early and subtle discomfort and edema.

## Data Availability

The raw data supporting the conclusions of this article will be made available by the authors, without undue reservation.
